# Identification through action potential clamp of proarrhythmic consequences of the short QT syndrome T618I hERG ‘hotspot’ mutation

**DOI:** 10.1016/j.bbrc.2022.01.057

**Published:** 2022-03-12

**Authors:** Chunyun Du, Henggui Zhang, Stephen C. Harmer, Jules C. Hancox

**Affiliations:** a School of Physiology, Pharmacology and Neuroscience, Biomedical Sciences Building, University Walk, Bristol, BS8 1TD, UK; b Biological Physics Group, Department of Physics and Astronomy, The University of Manchester, Manchester, M13 9PL, UK

**Keywords:** Arrhythmia, hERG, I_Kr_, Rapid delayed rectifier, Short QT syndrome, SQTS

## Abstract

The T618I *KCNH2*-encoded hERG mutation is the most frequently observed mutation in genotyped cases of the congenital short QT syndrome (SQTS), a cardiac condition associated with ventricular fibrillation and sudden death. Most T618I hERG carriers exhibit a pronounced U wave on the electrocardiogram and appear vulnerable to ventricular, but not atrial fibrillation (AF). The basis for these effects is unclear. This study used the action potential (AP) voltage clamp technique to determine effects of the T618I mutation on hERG current (I_hERG_) elicited by APs from different cardiac regions. Whole-cell patch-clamp recordings were made at 37 °C of I_hERG_ from hERG-transfected HEK-293 cells. Maximal I_hERG_ during a ventricular AP command was increased ∼4-fold for T618I I_hERG_ and occurred much earlier during AP repolarization. The mutation also increased peak repolarizing currents elicited by Purkinje fibre (PF) APs. Maximal wild-type (WT) I_hERG_ current during the PF waveform was 87.2 ± 4.5% of maximal ventricular repolarizing current whilst for the T618I mutant, the comparable value was 47.7 ± 2.7%. Thus, the T618I mutation exacerbated differences in repolarizing I_hERG_ between PF and ventricular APs; this could contribute to heterogeneity of ventricular-PF repolarization and consequently to the U waves seen in T618I carriers. The comparatively shorter duration and lack of pronounced plateau of the atrial AP led to a smaller effect of the T618I mutation during the atrial AP, which may help account for the lack of reported AF in T618I carriers. Use of a paired ventricular AP protocol revealed an alteration to protective I_hERG_ transients that affect susceptibility to premature excitation late in AP repolarization/early in diastole. These observations may help explain altered arrhythmia susceptibility in this form of the SQTS.

## Highlights

• T618I is a ‘hotspot’ hERG potassium channel mutation in the congenital short QT syndrome.
• Differences in hERG current during ventricular and Purkinje fibre action potentials are exacerbated by the T618I mutation.
• T618I has more modest effects on current during atrial action potentials.
• T618I modifies the protective response of hERG to premature ventricular excitation.
• These alterations to hERG function help explain ECG changes reported in T618I-hERG carriers.

## 1 Introduction

The speed of ventricular action potential (AP) repolarization determines the duration of the QT interval on the electrocardiogram. The integrated activity of a number of potassium channels, including those responsible for the rapid delayed rectifier current, I_Kr_, drives the repolarization process [1]. *human Ether-à-go-go Related Gene* (*hERG*; alternative nomenclature *KCNH2*) gives rise to the channels that underlie I_Kr_ [2,3]. It is now well established that loss-of-function *hERG* mutations are responsible for the LQT2 form of congenital long QT syndrome (LQTS) [4]. By contrast, gain-of-function *hERG* mutations lead to abbreviated ventricular repolarization and to the SQT1 form of the short QT syndrome (SQTS [5,6]). Although it is rare, the SQTS is clinically significant due to the fact that it is associated with an increased risk of ventricular and atrial arrhythmias and of sudden cardiac death [[5], [6], [7]]. Indeed, it is striking that cardiac arrest has been reported to be the most frequent presenting symptom of SQTS (in ∼40% of probands) [7].

It is notable that of the known SQTS ion channel mutations, SQT1 *hERG* mutations account for >50% of identified probands [5]. The T618I mutation, located at a highly conserved site in the channel pore-loop is of particular note because it has been ascribed “hotspot” status [[8], [9], [10]], accounting for ∼26% of genotyped probands [5,8,9]. This mutation has been found to occur in unrelated, geographically dispersed families (Europe, USA, Canada, China, Japan) [9], with a mean rate corrected (QT_c_) interval for probands and other carriers of 313 ms, poor rate adaptation of the QT_c_ interval, tall peaked T-waves, no gender preference in terms of carriers, 100% penetrance and high vulnerability to ventricular tachycardia and fibrillation [9]. A distinct U wave has been reported to be present in precordial leads of ∼70% of carriers [9]. However, in contrast to another prominent SQT1 hERG mutation (N588K), no reported T618I probands or carriers have experienced atrial fibrillation (AF; [9]). The reason for this is not understood. Biophysical investigations of the consequences of the T618I mutation for hERG channel current (I_hERG_) are in agreement that the mutation produces a gain-of-function effect, with changes to activation and inactivation gating reported [8,9,11,12]. However, there are disagreements between studies as to whether I_hERG_ activation is negatively [8,9] or positively [11,12]shifted by this mutation and the different experimental conditions used in these studies highlight the importance of characterising the mutation's effects under physiologically relevant conditions. Previous work on the N588K-hERG SQT1 mutation, using the AP voltage-clamp technique at physiological temperature has highlighted the possibility of differences in the extent of gain-of-function effect observed between APs from different regions of the heart [13]. The present study was undertaken to determine at physiological temperature the effect of the T618I mutation on I_hERG_ elicited by action potential (AP) waveforms from different cardiac regions (ventricle, Purkinje fibre (PF) and atria). The results obtained indicate that differences between ventricular and PF I_hERG_ during repolarization are exacerbated by the T618I mutation and that the effect of the mutation during atrial APs is attenuated compared to that during ventricular APs. These observations may help explain poorly understood aspects of the reported patient phenotype in this form of the SQTS.

## 2 Materials and methods

### 2.1 T618I hERG

Construction of the T618I mutation has been described previously [11]. Briefly, the following sense primer sequence was used in QuikChange ® (Agilent) mutagenesis: 5′CGG CGC TCT ACT TCA TCT TCA GCA GCC TCAC3′. DNA was sequenced across the entire insert (Eurofins MWG Operon) to confirm that only the desired mutation had been introduced.

### 2.2 Maintenance of cells and cell transfection

HEK-293 cells used for transient transfection were obtained from ECCAC (catalog number 85120602). Cells were passaged and maintained in culture as described previously [14,15]. 24–48 h after plating out, cells were transiently transfected with 0.5 μg of wild-type or T618I construct, using Lipofectamine™ 2000 (Invitrogen). Green Fluorescent Protein (GFP) was added as a transfection marker [16] at a ratio of 1:1. Cells were plated onto sterilized, collagen-coated glass coverslip shards. Recordings were made after at least 24 h incubation at 37 °C.

### 2.3 Electrophysiological recordings and solutions

Voltage clamp recordings were made at 37 ± 1 °C with a superfusate containing (in mM): 140 NaCl, 4 KCl, 2 CaCl_2_, 1 MgCl_2_, 10 Glucose, and 5 4-(2-hydroxyethyl)-1-piperazineethanesulfonic acid (HEPES) (titrated to pH of 7.45 with NaOH). Patch pipettes were filled with a solution containing (in mM): 130 KCl, 1 MgCl_2_, 5 EGTA, 5 MgATP, and 10 HEPES (titrated to pH of 7.2 with KOH). Series resistance values lay between 2 and 5 MΩ and were compensated 60–80%. hERG current (I_hERG_) recordings were made in the whole-cell mode using an Axopatch 1D amplifier (Axon instruments) and a CV-4 1/100 head stage. Data were recorded via a Digidata 1200B interface (Axon Instruments, USA) and stored on the hard-disk of a Viglen computer. Data digitization rates were 10–25 kHz during all protocols and an appropriate bandwidth of 2-10 kHz was set on the amplifier. The action potential (AP) waveforms used for AP voltage clamp experiments have been used in prior investigations from our laboratory [13,14]. Currents elicited under AP clamp were corrected online for P/N leak using an interspersed p/4 protocol. Data acquisition and analysis were performed using pCLAMP (Axon Instruments), Excel 365, Origin (2018 b) and GraphPad Prism (8) Software. Data are presented as mean ± S.E.M; statistical comparisons were made using paired or unpaired *t* tests, or 1-way ANOVA with Tukey's post-test as appropriate.

## 3 Results and discussion

**Fig. 1:**
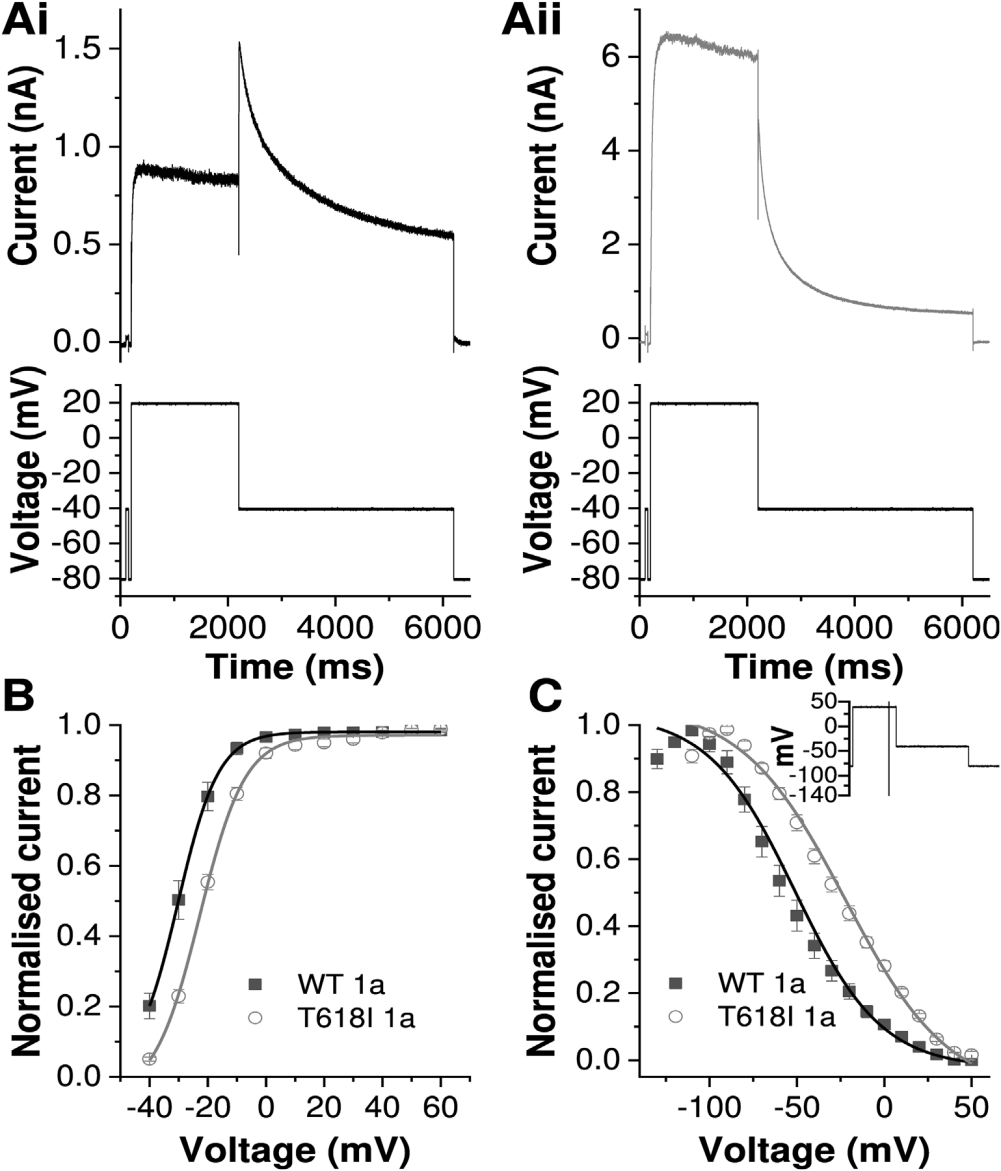
Effect of the T618I mutation on I_hERG_ profile during conventional voltage clamp **A.** Example current traces for WT (Ai) and T618I (Aii) I_hERG_ elicited by the protocol shown in the lower panels. Note that the current scales differ. **B.** Plots of voltage dependence of I_hERG_ activation for WT (black, n = 13) and T618I (grey, n = 11) hERG. These were derived from tail current (I_tail_) measurements on repolarization to −40 mV after depolarization commands between −40 and + 60 mV. I_tail_ values were normalized to the maximal I_tail_ elicited by the protocol and plotted against test command voltage. The plots were fitted by a Boltzmann function to give the V_0.5_ and *k* values in the main text. **C.** Voltage dependence of I_hERG_ inactivation plots for WT (black, n = 8) and T618I (grey, n = 7), elicited by a 3-step protocol shown as an inset. Currents elicited by the third step were normalized to maximal current elicited and plotted against preceding repolarization voltage. Data were fitted by a Boltzmann function to give the V_0.5_ and *k* values in the main text. Plotted values are uncorrected for deactivation. Both uncorrected and corrected V_0.5_ and *k* values are given in the main text.

Initial experiments characterised the alterations to I_hERG_ due to the T618I mutation under conventional voltage clamp. Fig. 1A shows representative recordings of WT and T618I I_hERG_ elicited by a command protocol comprised of a depolarization step to +20 mV and repolarization step to −40 mV [17,18]. WT I_hERG_ exhibited a typical resurgent tail current, with tail amplitude markedly greater than pulse current. By contrast, for T618I a much larger I_hERG_ was elicited during the +20 mV command than for the WT channel and tail current was markedly reduced compared to end-pulse current, as expected [8,9,11] (Fig. 1Aii). Additionally, the deactivation rate of T618I I_hERG_ was significantly faster than that of WT I_hERG_ (with deactivation t_half_ values 1198.0 ± 127.5 ms and 159.3 ± 9.9 ms for WT and T618I respectively, n = 12 for both WT and T618I, p < 0.0001, *t*-test). Through application of a protocol in which the command voltage of the 2s pulse was varied between −40 and + 60 mV [14,19], tail current analysis was performed to characterise voltage-dependent activation of T618I I_hERG_ (Fig. 1B). For WT I_hERG_ the mean activation V_0.5_ obtained was −28.7 ± 1.1 mV (*k* = 5.3 ± 0.7, n = 13), whilst for T618I I_hERG_ this was −22.9 ± 0.7 mV (*p* < 0.001 vs WT; *k* = 7.9 ± 0.5, p < 0.01 vs WT, n = 11). The modest *positive* shift in voltage-dependent activation is in qualitative agreement with prior studies conducted at physiological/near physiological temperature [11,12]. Notably, it is incompatible with a contribution of *negatively* shifted voltage dependent activation to the gain-of-function effect of T618I reported from room temperature measurements [9]. Fig. 1C compares voltage dependent inactivation between WT and T618I I_hERG_. The 3-step protocol used is shown as an inset to Fig. 1C (see also [11,14,19]). The I_hERG_ inactivation V_0.5_ was positively shifted by the T618I mutation (WT V_0.5_ = −55.4 ± 4.9 mV, *k* = 21.2 ± 1.4, n = 8; T618I V_0.5_ = −21.5 ± 4.4 mV, *k* = 30.4 ± 1.6, n = 7; p < 0.001 vs WT for both V_0.5_ and *k* (cf [8,11])). When the data were corrected for potential deactivation induced by the brief repolarizing step phase of the protocol (as in [19]), WT V_0.5_ was −61.8 ± 5.0 mV, *k* = 22.8 ± 1.8, n = 8, and T618I V_0.5_ was −37.4. ± 6.7 mV, *k* = 25.7 ± 2.6, n = 7; p < 0.05 vs WT for V_0.5_ and p = 0.37 for *k*). In summary, at 37 °C the T618I mutation produced positive voltage shifts in both activation and inactivation, but with a more marked effect on the latter.

**Fig. 2:**
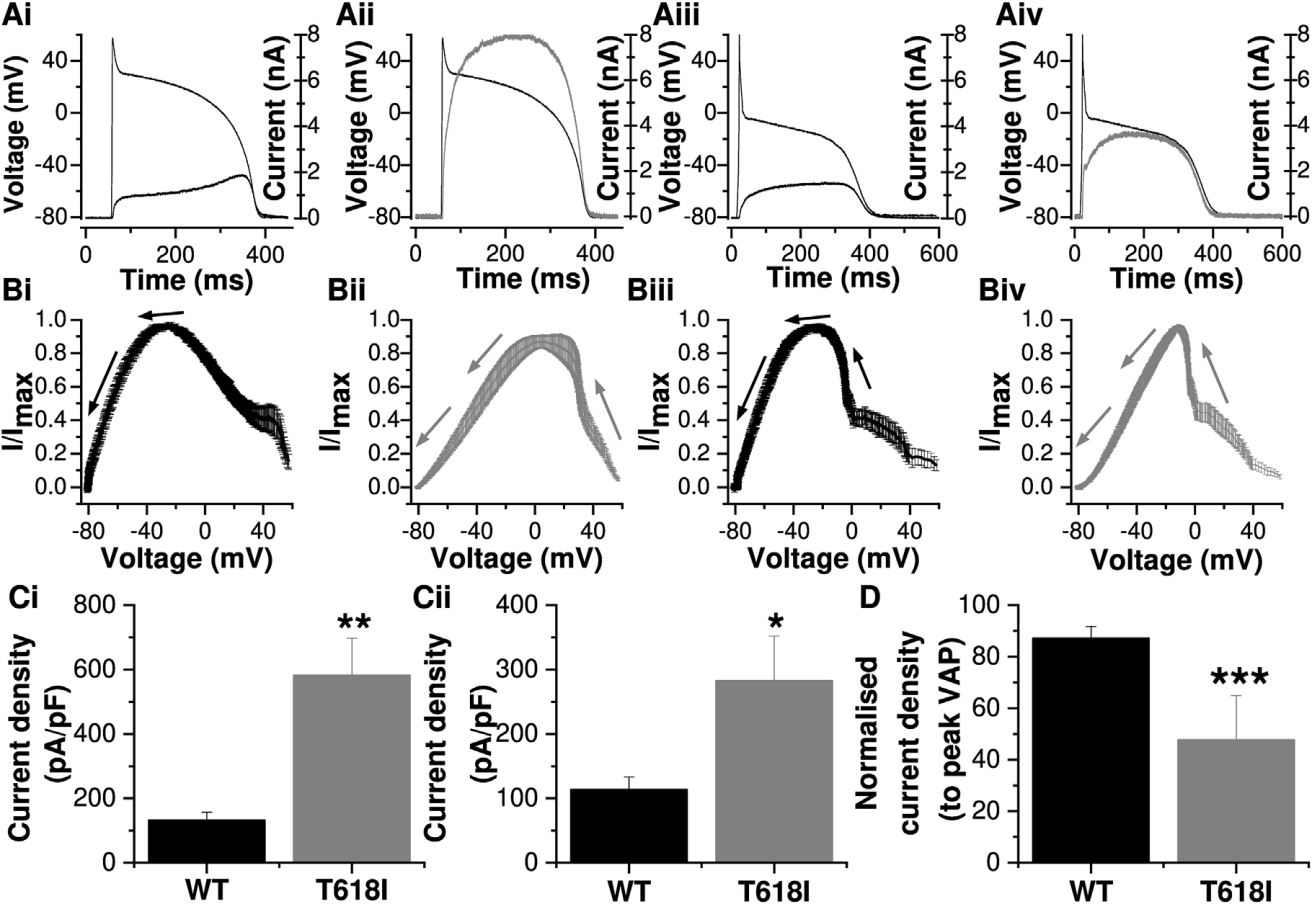
I_hERG_ during ventricular and Purkinje fibre (PF) AP waveforms **A.** Ventricular AP command waveform overlaid with WT (Ai) and T618I (Aii) I_hERG_ profiles. Purkinje fibre (PF) AP command waveform overlaid with WT (Aiii) and T618I (Aiv) I_hERG_ profiles. **B.** Normalized instantaneous current–voltage (I–V) relations for WT (Bi) and T618I (Bii) I_hERG_ during ventricular AP repolarization and for WT (Biii) and T618I (Biv) I_hERG_ during PF AP repolarization. Currents were normalized to maximal current during AP repolarization. The arrows show the direction of AP repolarization (n = 7 for WT and n = 8 for T618I). **C**. Bar charts showing the mean (±SEM) peak I_hERG_ density during repolarization phase of ventricular AP (Ci) and PF AP (Cii) for WT (black, n = 7) and T618I (grey, n = 8). **D**. Plots of the normalized peak I_hERG_ density during PF AP for WT (black) and T618I (grey) hERG. The peak I_hERG_ density during PF AP was normalized to that during ventricular AP from the same cell. ”∗” denotes statistical significance of p < 0.05; “∗∗” denotes statistical significance of p < 0.01; “∗∗∗” denotes statistical significance of p < 0.001. unpaired *t*-test.

Fig. 2 shows the profile of I_hERG_ during ventricular and Purkinje fibre (PF) AP waveforms. For WT I_hERG_, current increased progressively during the plateau phase peaking late in the AP, prior to the terminal repolarization phase (Fig. 2Ai, Bi; [[19], [20], [21]]). A plot of the mean normalized instantaneous current-voltage (I–V) relationship showed that maximal I_hERG_ occurred at −27.0 ± 2.7 mV (n = 7). For T618I expressing cells, I_hERG_ during the AP increased progressively following the AP upstroke and during the early part of the plateau, then levelling out prior to declining during the latter part of the AP (Fig. 2Aii). The mean instantaneous I–V relation (Fig. 2Bii) showed that maximal I_hERG_ occurred much earlier during AP repolarization (11.4 ± 5.3 mV (n = 8; p < 0.001 vs WT). This inverted U or bow-shaped current profile is similar to that reported previously for T618I hERG during ventricular APs at physiological temperature [11], but differs from that reported at room temperature [9]. Fig. 2Ci shows mean peak repolarizing current density during the ventricular AP command for WT and T618I channels, with that for the SQT1 mutant being ∼4-fold the WT amplitude.

The PF AP command waveform used for this study had prominent phase 1 repolarization and consequently a lower plateau phase than that present in the ventricular AP command (cf [13,21]). As a consequence, WT I_hERG_ increased less steeply during the AP plateau than observed with the ventricular AP command (Fig. 2Aiii), with maximal current during repolarization peaking at −21.5 ± 2.4 mV (Fig. 2Biii; n = 7). T618I I_hERG_ elicited by the PF AP exhibited (Fig. 2Aiv) a similar inverted U-shaped profile to that elicited by the ventricular AP, with maximal current during repolarization occurring at a more positive potential (Fig. 2Biv −11.2 ± 1.3 mV; n = 8) than for the WT channel (p < 0.01). Fig. 2Cii shows mean peak repolarization current density during the PF command for WT and T618I channels, with peak T618I I_hERG_ nearly 3-fold that for the WT channel. A previous study of the N588K SQT1 mutation [13], which has a more severe attenuation of voltage-dependent inactivation [19,22] than exhibited by T618I, reported exacerbation of differences between ventricular and PF peak repolarizing I_hERG_ by the SQT1 mutation. We investigated whether or not this may be the case for the T618I mutation by expressing peak repolarizing current during the PF command as a proportion (percentage) of that elicited by the ventricular AP command, with both commands applied in the same experiment. The bar chart in Fig. 2D shows the results of this analysis. For WT I_hERG_ peak current elicited by the PF command was 87.2 ± 4.5% of that elicited by the ventricular AP command (n = 7). In contrast, for T618I I_hERG,_ peak current elicited by the PF command was 47.7 ± 2.7% of that elicited by the ventricular AP command (n = 8; p < 0.001). Thus, ventricular-PF differences in peak repolarizing I_hERG_ were augmented by the T618I mutation. This difference could contribute to heterogeneity of effect of the T618I mutation on ventricular and PF repolarization which, in turn, could account for or contribute to the pronounced ‘U’ waves seen on the ECG of the majority of T618I mutation carriers [9].

**Fig. 3:**
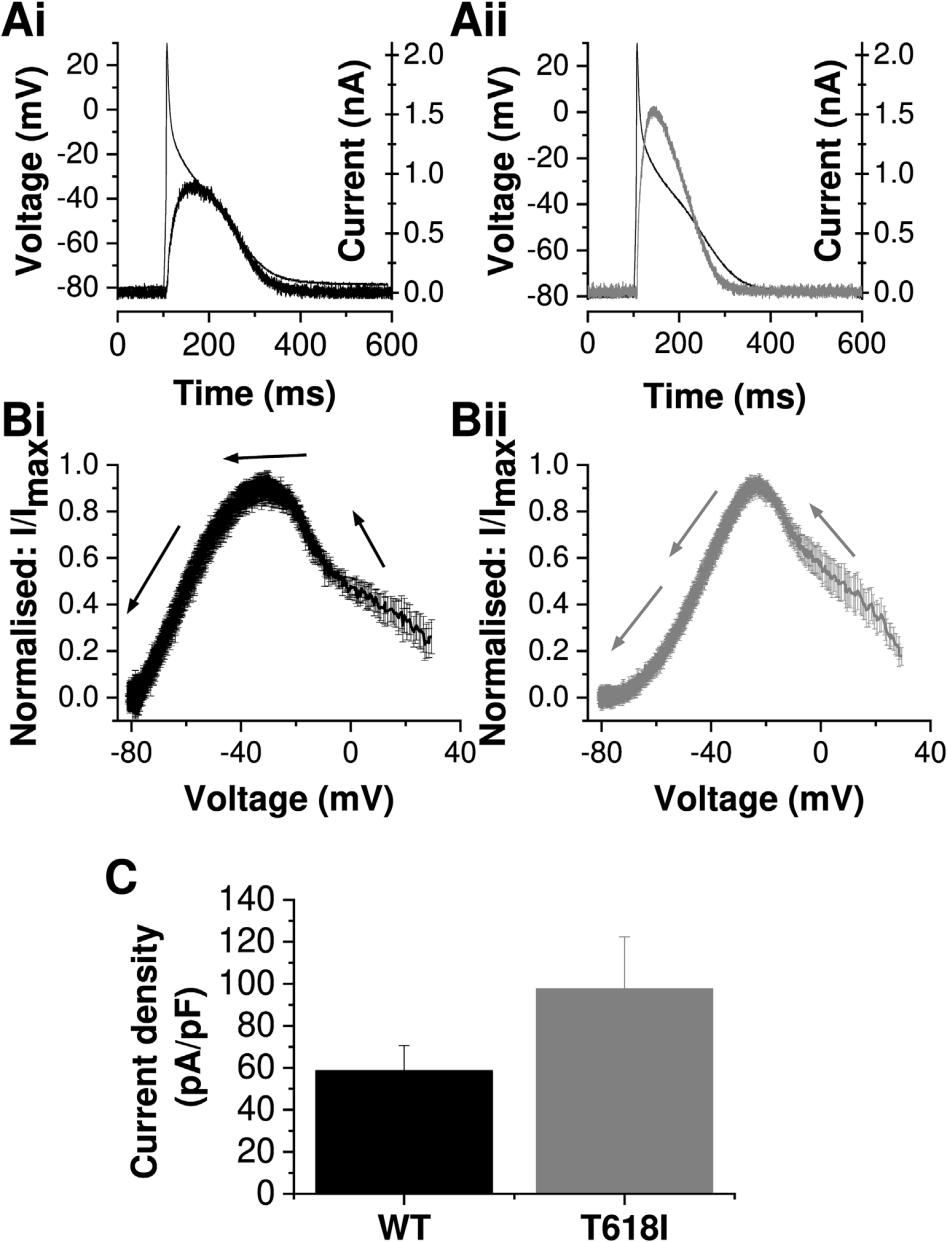
I_hERG_ profile under atrial AP clamp **A**. Atrial AP command waveform overlaid with WT (Ai) and T618I (Aii) I_hERG_ profiles. **B**. Normalized instantaneous current–voltage (I–V) relations for WT (Bi, n = 7) and T618I (Bii, n = 8) I_hERG_ during atrial AP repolarization. Currents were normalized to maximal current during AP repolarization. The arrows show the direction of AP repolarization. **C.** Bar charts showing the mean (±SEM) WT (black, n = 7) and T618I (grey, n = 8) peak I_hERG_ density during repolarization phase of atrial AP (p = 0.2, unpaired *t*-test).

Fig. 3 shows the profiles of WT and T618I I_hERG_ under atrial AP clamp conditions. The relatively brief AP duration and absence of a distinct plateau phase resulted in I_hERG_ under WT and mutant conditions (Fig. 3Ai and Aii) that differed from their counterparts during the ventricular and PF AP commands (Fig. 2). First, the peak outward repolarizing current was smaller for each of WT and T618I during the atrial AP command than during either ventricular or PF commands (compare Fig. 3C with Fig. 2C). Second, although there was a trend for the I_hERG_ magnitude to be larger for T618I than WT I_hERG_ during the atrial AP command (Fig. 3Ai, Aii and B), when mean peak repolarizing I_hERG_ density was compared between WT and T618I conditions the difference did not attain statistical significance (Fig. 3B; p > 0.1). Third, as indicated for the raw current traces in Fig. 3A and instantaneous I–V plots in Fig. 3B, the profiles of WT and T618I I_hERG_ during the atrial AP were similar to one-another, albeit with a moderate positive shift in the voltage at which peak repolarizing I_hERG_ occurred (from −30.0 ± 2.0 mV for WT (n = 7) to −20.3 ± 1.4 mV for T618I (n = 8; p < 0.01 vs WT)). The reduced effect of the T618I mutation on I_hERG_ during the atrial AP than during either ventricular or PF APs may account for the lack of atrial fibrillation seen in T618I carriers [8,9].

**Fig. 4:**
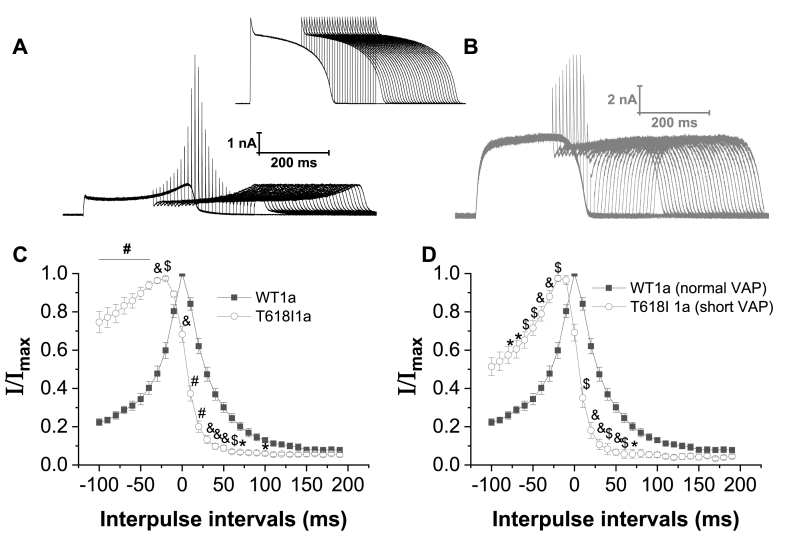
Effect of T618I mutation on I_hERG_ transients with paired AP stimulation **A**. Example of families of WT I_hERG_ elicited by a protocol comprised of paired ventricular AP command waveforms shown in the inset between A and B. **B**. Example of families of T618I I_hERG_ elicited by paired ventricular AP command waveforms shown in the inset between A and B. **C**. Plots of the peak outward current transients during the paired ventricular AP command waveforms against interpulse interval for WT (black, n = 7) and T618I (grey, n = 10) I_hERG_. The I_hERG_ transients were normalized to the peak I_hERG_ transient amplitude observed in each individual cell. **D**. Similar plots as panel C, with WT I_hERG_ current transients elicited with normal ventricular AP (black, n = 7) and T618I I_hERG_ elicited with abbreviated (APD_90_ shortened by 46%; n = 5) ventricular AP. In panels C and D, ‘0’ on the time axis denotes the APD_90_ point of the first of the pair of APs. “#” denotes statistical significance of p < 0.0001; “&” denotes statistical significance of p < 0.001; “$” denotes statistical significance of p < 0.01; “∗” denotes statistical significance of p < 0.05; (two-way ANOVA with Sidak's multiple comparisons test).

In addition to regulating AP repolarization, due to its unique kinetics the hERG/I_Kr_ channel can generate rapid outward transient currents that are protective against premature excitation late in AP repolarization/early in diastole [23,24]. Pathological mutations can modify this protective role [13,24]. Therefore, additional experiments were conducted to investigate effects of the T618I mutation on outward transient currents generated by application of a second AP waveform applied late in repolarization or early following completion of repolarization. The protocol used is shown as an inset above Fig. 4A and B, with representative currents for WT and T618I I_hERG_ shown respectively in Fig. 4A and B. A second ventricular AP command was applied at different intervals ranging from −100 ms to +190 ms in relation to APD_90_ of the initial AP command. As illustrated by the representative currents in Fig. 4A and B, the pattern of rapid current transients elicited by the protocol differed between WT and T618I conditions. The time-dependent profile of the transient currents was obtained by normalizing transient amplitude elicited by the second AP of the pair at each interpulse interval to the maximal transient current amplitude observed. The resulting mean values were plotted against interpulse interval, with APD_90_ ascribed a value of zero [13,25]. Fig. 4C shows transient time-course superimposed for WT and T618I I_hERG._ Between −100 and −20 ms before APD_90_ T618I normalized I_hERG_ transients were greater than those for WT I_hERG_. However, at APD_90_ and thereafter T618I I_hERG_ transients declined more rapidly than those for WT I_hERG_, consistent with a potentially reduced protective role late in repolarization/early in diastole. This phenomenon was studied further by performing additional paired AP experiments on T618I I_hERG_ in which an abbreviated ventricular AP (APD_90_ shortened by 46% cf [13]) was applied to approximate an SQTS condition (ie. hastened repolarization). The normalized profile of T618I I_hERG_ transients with paired ‘short’ action potentials was then overlaid that for WT I_hERG_ with the non-abbreviated AP command protocol (Fig. 4D). The overall result was similar to that shown in Fig. 4C: the normalized transients declined more quickly for the mutant than WT channel. Accelerated deactivation of T618I I_hERG_ is likely to contribute to this change. The change in response to premature AP commands is consistent with an altered susceptibility to unwanted premature stimulation in the T618I setting.

Several conclusions can be made from the results of this study. First, the positively shifted voltage dependent activation observed here at physiological temperature is incompatible with a prior suggestion from room temperature data that negatively shifted activation contributes to the gain-of-function mechanism of the T618I mutation [9]. This underscores the importance of studying clinical channel variants at physiological temperature. Positively shifted inactivation (Fig. 1 [8,11],) is likely key to the gain-of-function phenotype in this SQT1 variant. Second, regional differences in AP configuration – in particular the presence and height of the plateau phase - are likely to feed into the consequences of the T618I mutation. These regional differences led to an augmented ventricular-PF difference in repolarizing I_hERG_, which may contribute to heterogeneity of repolarization (evident in the U wave of T618I carriers [9]) and to a substrate for re-entry. They likely also account for the greatly reduced effect of the mutation during the atrial AP command used in this study. Finally, our results also highlight that the T618I mutation may reduce the channel's ability to generate protective current transients early in diastole, which could lead to increased susceptibility to unwanted premature stimulation. Future work using human cell and tissue models that incorporate regional differences in AP configuration are now warranted to interrogate how the findings of this study impact arrhythmia substrates in the setting of intact multicellular cardiac tissue.

## Declaration of competing interest

The authors declare no conflicts of interest.
